# German Cardiac Arrest Registry (G-CAR)—results of the pilot phase

**DOI:** 10.1007/s00392-024-02468-5

**Published:** 2024-06-13

**Authors:** Janine Pöss, Christoph Sinning, Michelle Roßberg, Nadine Hösler, Taoufik Ouarrak, Bernd W. Böttiger, Sebastian Ewen, Harm Wienbergen, Fabian Voss, Jochen Dutzmann, Eike Tigges, Ingo Voigt, Anne Freund, Steffen Desch, Guido Michels, Holger Thiele, Uwe Zeymer, the G-CAR Investigators

**Affiliations:** 1 Leipzig Heart Center, Leipzig, Germany; 2 University Heart & Vascular Center Hamburg, Hamburg, Germany; 3 Leipzig Heart Science, Leipzig, Germany; 4 Institut Für Herzinfarktforschung, Ludwigshafen, Germany; 5 Faculty of Medicine and University Hospital Cologne, Cologne, Germany; 6 University Hospital Saarland, HomburgSaar, Germany; 7 Klinikum Links Der Weser, Bremen, Germany; 8 University Hospital Düsseldorf, Düsseldorf, Germany; 9 University Hospital Halle (Saale), Halle, Germany; 10 Asklepios Clinic Sankt Georg Hamburg, Hamburg, Germany; 11 Elisabeth Hospital Essen, Essen, Germany; 12 Krankenhaus Der Barmherzigen Brüder, Trier, Germany; 13 Klinikum Ludwigshafen, Ludwigshafen, Germany

**Keywords:** Cardiopulmonary resuscitation (CPR), Out-of-hospital cardiac arrest (OHCA), Registry, Extracorporeal cardiopulmonary resuscitation (eCPR), Cardiac arrest centre (CAC), Post-resuscitation care

## Abstract

**Background:**

In Europe, more than 300,000 persons per year experience out-of-hospital cardiac arrest (OHCA). Despite medical progress, only few patients survive with good neurological outcome. For many issues, evidence from randomized trials is scarce. OHCA often occurs for cardiac causes. Therefore, we established the national, prospective, multicentre German Cardiac Arrest Registry (G-CAR). Herein, we describe the first results of the pilot phase.

**Results:**

Over a period of 16 months, 15 centres included 559 consecutive OHCA patients aged ≥ 18 years. The median age of the patients was 66 years (interquartile range 57;75). Layperson resuscitation was performed in 60.5% of all OHCA cases which were not observed by emergency medical services. The initial rhythm was shockable in 46.4%, and 29.1% of patients had ongoing CPR on hospital admission. Main presumed causes of OHCA were acute coronary syndromes (ACS) and/or cardiogenic shock in 54.8%, with ST-elevation myocardial infarction being the most common aetiology (34.6%). In total, 62.9% of the patients underwent coronary angiography; percutaneous coronary intervention (PCI) was performed in 61.4%. Targeted temperature management was performed in 44.5%. Overall in-hospital mortality was 70.5%, with anoxic brain damage being the main presumed cause of death (38.8%). Extracorporeal cardiopulmonary resuscitation (eCPR) was performed in 11.0%. In these patients, the in-hospital mortality rate was 85.2%.

**Conclusions:**

G-CAR is a multicentre German registry for adult OHCA patients with a focus on cardiac and interventional treatment aspects. The results of the 16-month pilot phase are shown herein. In parallel with further analyses, scaling up of G-CAR to a national level is envisaged.

Trial registration

ClinicalTrials.gov identifier: NCT05142124.

## Introduction

In Europe, more than 300,000 patients suffer from out-of-hospital cardiac arrest (OHCA) every year [1]. Despite medical progress leading to a trend towards decreased mortality rates of OHCA patients [2, 3], reported survival rates with good neurological outcome remain low, ranging from 2 to 10% [4–6]. Therefore, optimization of medical care of these patients is necessary. However, adequate evidence from randomized trials is scarce and clinical practice differs widely between centres. Against this background, a systematic and standardized registration of the treatment course and of the clinical outcomes of OHCA patients in a “real-world setting” is essential and has become a prerequisite for the certification of cardiac arrest centres in Germany [7]. Despite the fact that cardiac pathologies are the underlying cause in the majority of OHCA cases [8], a national registry including long-term and patient-reported outcomes, such as health-related quality of life, psychopathological symptoms (i.e. cognitive impairment, depression, anxiety, or post-traumatic stress disorder) and return to normal life under the guidance of cardiologists has not been published so far concerning the German population. As previously described in more detail [9], we established G-CAR (German Cardiac Arrest Registry) with the aim to achieve a better understanding of the acute and long-term consequences of OHCA and to optimize processes during treatment and follow-up of OHCA patients. Herein, we report the results of the pilot phase of this new registry with a special focus on interventional aspects, such as treatment of coronary artery disease and extracorporeal cardiopulmonary resuscitation (eCPR).

## Methods

### Design and overview

G-CAR (NCT05142124) is a prospective, multicentre registry of patients with OHCA in Germany. The design of the trial was published previously [9]. In total, 15 centres agreed to participate in the pilot phase and to provide data on their patients by filling out the electronic case report form. The registry collects information regarding the (i) pre-hospital phase; (ii) in-hospital phase including interventional therapy, treatment on the intensive care unit (ICU), and eCPR; and (iii) post-hospital (rehabilitation) phase. The collected information is stored at the Institut für Herzinfarktforschung in Ludwigshafen, Germany.

### Patients and follow-up

Patients ≥ 18 years with OHCA were eligible for inclusion. The only exclusion criterion was rejection of participation by either the patient or the legal representative. Patients were treated according to the local standards of the participating centres. Clinical and neurological outcomes were and will be assessed after 30 days, 6 months, and 1 year. The evaluation after 30 days is performed either by a personal interview if the patient is still hospitalized or by a structured telephone interview by qualified staff. After 6 and 12 months, patient-reported outcomes such as health-related quality of life, psychopathological symptoms (cognitive impairment, affective disorders, post-traumatic stress disorder), and social reintegration are assessed by standardized questionnaires and by a telephone interview using standardized questionnaires.

### Informed consent

Due to their clinical status, some patients were unable to give consent on admission to the hospital. Patients who were able to give consent during the hospital stay were asked to do so as soon as possible. If patients were unable to give informed consent during the hospital stay, her/his consent was sought from their legal representative. Data of patients who died before regaining their capacity to consent and before a legal representative was appointed were anonymized and included in the registry. This informed consent process has been validated and approved by the ethical committees in all federal counties of the recruiting study centres in Germany.

## Results

**Fig. 1:**
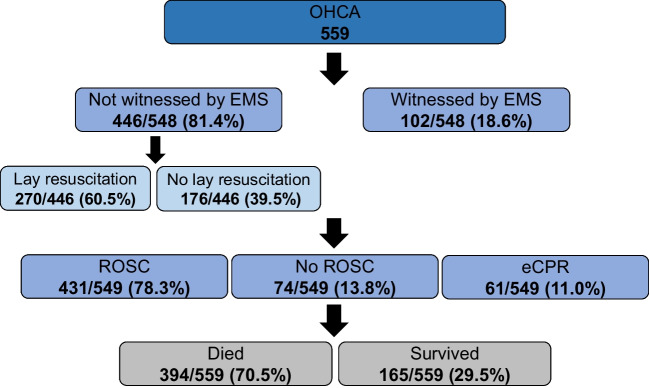
Flow diagram of study patients. OHCA, out-of-hospital cardiac arrest; ROSC, return of spontaneous circulation; (e)CPR, (extracorporeal) cardiopulmonary resuscitation. Due to missing information about the different variables, the total number of patients differs within the figure

A total of 559 consecutive adult OHCA patients were included in 15 tertiary care hospitals between 07/2021 and 03/2023. Out of the 15 centres involved in the pilot phase, 13 (86.7%) are certified cardiac arrest centres. The baseline characteristics of the patients are depicted in Table 1. The median age of the patients was 66 years (interquartile range [IQR] 57;75), and 73.7% were male. The medical history of the patients encompasses the classical comorbidities, such as a history of myocardial infarction (17.0%), peripheral artery disease (9.6%), and chronic kidney disease (12.0%). Figure 1 depicts a flow diagram of the included patients.

**Table 1:** Baseline characteristics

Number of patients	559
Age	66 (57;75)
Male	73.7% (412/559)
Medical history	
Myocardial infarction	17.0% (73/430)
Coronary artery bypass grafting	5.6% (25/447)
Stroke	6.7% (29/435)
Peripheral artery disease	9.6% (42/437)
Chronic kidney disease	12.0% (53/441)
Chronic obstructive pulmonary disease	8.2% (36/438)

Variables are represented as median (interquartile range) or percentage (frequency)

### Pre-hospital phase

Mostly, OHCA occurred in private homes (52.2%), followed by public spaces in around one-third of cases (32.5%). Less frequently, patients suffered OHCA in medical facilities (6.3%), nursing homes (5.3%), or workspaces (3.7%). OHCA was unwitnessed in 22.1%, witnessed by laypersons in 59.3%, and by emergency medical services (EMS) in 18.6%. CPR was dispatcher-assisted in 11.5%; automatic external defibrillators (AED) were used in 7.2%, and a first-responder App in 0.7% of cases. Layperson resuscitation was performed in 60.5% of all OHCA cases which were not observed by EMS. The initial rhythm was shockable in 46.4%, non-shockable in 46.0%, and not documented in 7.6% (Table 2).

**Table 2:** Pre-hospital phase

Location of CPR	
Public	32.5% (177/544)
Nursing home	5.3% (29/544)
Private home	52.2% (284/544)
Workspace	3.7% (20/544)
Medical facility	6.3% (34/544)
CPR details	
Dispatcher-assisted CPR	11.5% (62/539)
Public access defibrillator	7.2% (39/544)
First-responder App used	0.7% (4/546)
Collapse	
Witnessed	77.9% (427/548)
Unwitnessed	22.1% (121/548)
Layperson resuscitation*	
No	39.5% (176/446)
Yes	60.5% (270/446)
Initial rhythm	
Shockable (VT/VF)	46.4% (256/552)
Non-shockable (Asystole/PEA)	46% (254/552)
Not reported	7.6% (42/552)
Other characteristics and time intervals	
Ventilation at admission	87.4% (408/467)
Another episode of CPR after ROSC	26.0% (123/473)
Low-flow (total CPR) time, min	29 (15; 57)

Variables are represented as percentage (frequency)

*CPR*, cardiopulmonary resuscitation; *EMS*, emergency medical services; *VF*, ventricular fibrillation; *VT*, ventricular tachycardia; *PEA*, pulseless electrical activity; *ROSC*, return of spontaneous circulation

^*^Layperson resuscitation rate reported according to the new calculation of the German Resuscitation Registry, referring only to OHCA cases unwitnessed by the EMS

### In-hospital phase

The majority of patients (64.1%) were admitted to a shock room or an emergency department, 24.3% were directly transferred to the catheterization laboratory, and 11.6% were admitted to an ICU. In total, 29.1% of the patients had ongoing CPR on hospital admission (Table 2).

**Fig. 2:**
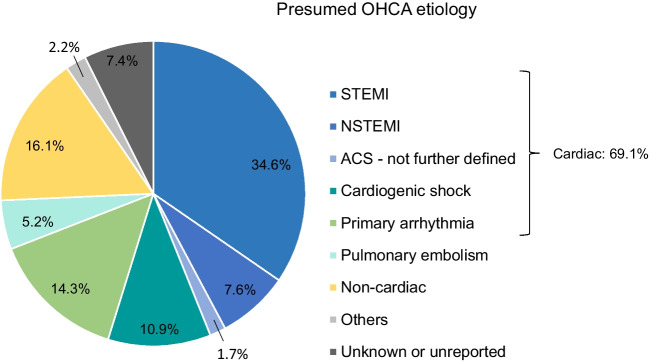
Presumed aetiologies of OHCA

Main presumed underlying causes of OHCA were acute coronary syndromes (ACS) and/or cardiogenic shock (CS) in more than half (54.8%) of the patients. ST-elevation myocardial infarction was observed in more than one-third (34.6%) of the cases. Primary arrhythmia without suspected ischemia was reported in 14.3%. Overall, the presumed cause for OHCA was cardiac in 69.1%. Non-cardiac causes included pulmonary embolism in 5.2%, and other non-cardiac causes (i.e. intoxication, hypoxia, hypovolemia, hypo-/hyperkalaemia, hypothermia, pneumothorax, and neurological causes) in 16.1% (Fig. 2).

### Coronary angiography (CAG)

**Fig. 3:**
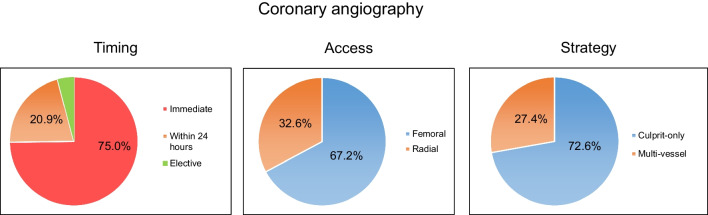
Coronary angiography data: **A** timing of coronary angiography, **B** access, **C** strategy (PCI, percutaneous coronary intervention)

**Fig. 4:**
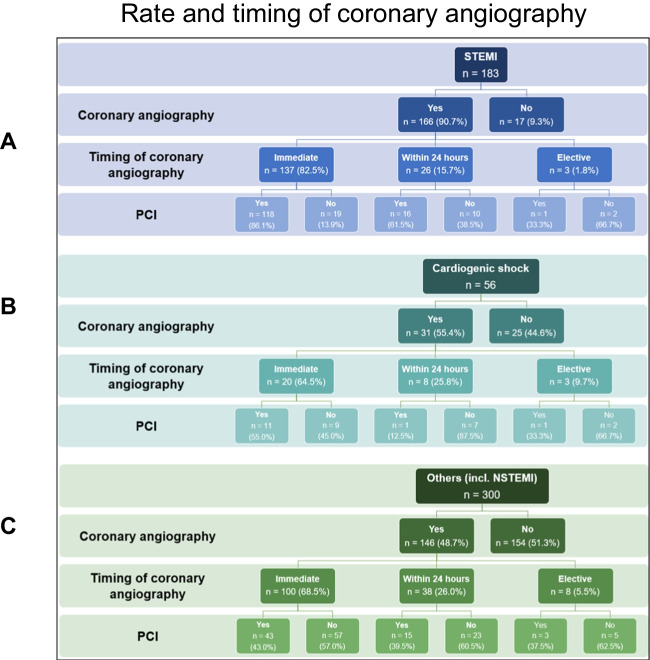
Rate and timing of coronary angiography according to the presumed underlying cause of OHCA: **A** STEMI, **B** cardiogenic shock, **C** all other aetiologies (including NSTEMI, excluding STEMI and cardiogenic shock) (NSTEMI, non-ST-elevation myocardial infarction; PCI, percutaneous coronary intervention; STEMI, ST-elevation myocardial infarction). Note: **A** Only 167/169 patients with available information on timing of coronary angiography and PCI. **C** Only 138/141 patients with available information on timing of coronary angiography and PCI

Figure 3 A–C depicts the relevant findings regarding coronary angiography. In total, 62.9% of the patients underwent CAG. In three-quarters (75.0%) of these patients, CAG was performed immediately, and in 20.9% within 24 h. The remaining patients underwent elective CAG. Out of all patients undergoing CAG, 61.2% received percutaneous coronary intervention (PCI). Figure 4 depicts the rate and timing of CAG according to the presumed underlying cause of OHCA (STEMI (A), cardiogenic shock (B), and all other aetiologies [including NSTEMI, excluding STEMI and CS] (C)). More than two-thirds (67.2%) of the procedures were performed via femoral access. Culprit-only PCI was performed in 72.6% of cases, while immediate multivessel PCI was performed in 27.4%

### Targeted temperature management (TTM)

Targeted temperature management was performed in 44.5% (Table 3). Among those patients, therapeutic hypothermia was performed in 87.0% with a mean target temperature of 35.0 °C and a mean duration of 24 h. In 42.8%, TTM was applied invasively (i.e. intravascular catheter), in 49.0% non-invasively, and in 8.2% via an extracorporeal life support (ECLS) system. Active fever prevention was performed in 69.3% over a period of 72 h.

**Table 3:** Targeted temperature management

Temperature management	44.5% (245/551)
Hypothermia (32–36.5 °C)	85.6% (178/208)
Target temperature	
33 °C	30/208 14.4%
34 °C	59/208 28.4%
35 °C	84/208 40.4%
36 °C	35/208 16.8%
Method of cooling	
Invasive	42.8% (89/208)
Non-invasive	49.0% (102/208)
Via ECMO	8.2% (17/208)
Fever prevention	69.3% (124/179)

Variables are represented as median (interquartile range) or percentage (frequency)

*ECMO*, extracorporeal membrane oxygenation

### eCPR

**Fig. 5:**
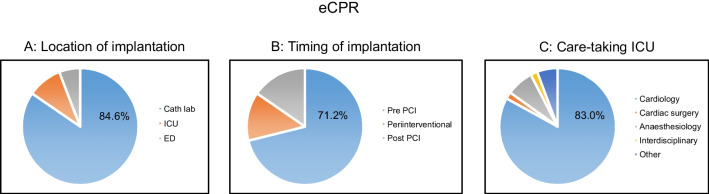
eCPR: **A** location of implantation, **B** timing of implantation, **C** care-taking ICU (eCPR, (extracorporeal) cardiopulmonary resuscitation; ICU, intensive care unit)

Relevant data regarding eCPR are depicted in Fig. 5A–C. eCPR was performed in 11.0% of the patients. Implantation of an ECLS system was mostly performed in the catheterization laboratory (84.6%), followed by the ICU (9.6%) and the emergency department (5.8%) (Fig. 5A). Coronary angiography was performed in 82%, and PCI in 59.2%. Most ECLS systems were implanted before PCI (71.2%), 13.5% during PCI, and 15.4% after PCI (Fig. 5B). Cannulation was performed percutaneously in 98.1%. In most of the cases (83.0%), the care-taking ICU had a cardiology specialisation, followed by anaesthesiology (7.5%), interdisciplinary (1.9%), or surgical (1.9%) ICUs (Fig. 5C). Complications of eCPR are depicted in Table 4. Bleeding complications occurred in one-third (33.3%) of patients and were mainly located at the access site. Limb ischemia was observed in 9.8%, and ischemic stroke in 2.0% of cases.

**Table 4:** Complications of extracorporeal CPR

Patients with eCPR (% of total, *n*/*n*)	11.0% (61/549)
Bleeding complications	**33.3% (17/51)**
Cannulation site	15.7% (8/51)
Cerebral	3.9% (2/51)
Other	13.7% (7/51)
Ischemic complications	**13.7% (7/51)**
Limb ischemia	9.8% (5/51)
Limb ischemia leading to amputation	2.0% (1/51)
Ischemic stroke	2.0% (1/51)

### Outcomes

Overall, the in-hospital mortality rate was 70.5% with anoxic brain damage representing the leading cause of death (38.8%), followed by circulatory (30.6%) and multiorgan failure (11.6%) (Table 5). In more than half of the patients (54.9%), in-hospital treatment was terminated prematurely. Main reasons for termination were the occurrence of anoxic brain damage (32.9%) and presumed patient will (28.2%) (Table 5). In-hospital mortality of eCPR patients was 85.2%.

**Table 5:** Outcomes

Discharged alive	29.5% (165/559)
CPC	
1	81.9% (68/83)
2	13.3% (11/83)
3	1.2% (1/83)
4	3.6% (3/83)
5	0
In-hospital mortality	**70.5% (394/559)**
Presumed cause of death	
Anoxic brain damage	38.3% (151/394)
Circulatory failure	30.2% (119/394)
Multiorgan failure	11.4% (45/394)
Others	18.7% (74/394)
Not specified	1.3% (5/394)
Termination of in-hospital treatment	**54.9% (303/552)**
Reason for termination	
Anoxic brain damage	32.7% (99/303)
Circulatory failure	21.1% (64/303)
Multiorgan failure	6.6% (20/303)
Patient will	28.1% (85/303)
Limiting comorbidity	3.6% (11/303)
Other	7.3% (22/303)
Not specified	0.7% (2/303)

## Discussion

Herein, we present the results of the 16-month pilot phase of G-CAR including 559 patients and covering the pre-hospital and in-hospital phases with a focus on interventional aspects, including data on coronary angiography and eCPR.

### Baseline data and pre-hospital phase

All 15 participating centres are tertiary care hospitals. The reported mean age of 66.0 years is comparable to the results of the European EuReCa TWO study (67.6 years) and slightly lower compared to the 2022 report of the German Resuscitation Registry (GRR; 70.2 years) [5]. The predominance of male sex has also been reported in the other two registries. As shown in these data, most OHCA cases occurred at home.

The rate of witnessed OHCA was higher in our cohort than in the GRR (77.9% vs. 56.3%, respectively). Correspondingly, the rate of a shockable initial rhythm was much higher (46.4% vs. 19.8%), and the rate of layperson CPR was higher (60.5% vs. 51.3%). Most likely, this is explained by the fact that the centres involved in G-CAR are mostly cardiac arrest centres with a focus on cardiological patients. Furthermore, most centres are located in urban areas with a high population density and a high number of potential bystanders. The higher rate of underlying cardiac causes in G-CAR compared to the GRR (69.1% vs. 57.9%, respectively) and the high rate (54.8%) of patients presenting with an ACS or with CS as presumed underlying aetiology in G-CAR might explain this difference. Unfortunately, the GRR does not further classify cardiac causes, making a direct comparison of the incidence of ACS as underlying cause impossible [10].

The rate of dispatcher-assisted CPR was lower in our registry compared to the GRR (11.5% vs. 30.8%, respectively). The reasons for this are unclear and might be partly due to a reporting-bias. Clearly, the dispatcher-assisted CPR in Germany needs to be further developed [11]. The reported rate of AED use was higher in our cohort compared to the GRR (7.2% vs. 1.5%, respectively). Notably, a first-responder App was used in only 0.7% of the cases. Compared with other countries, e.g. Denmark [12, 13], this is extremely low and needs to be improved.

### Coronary angiography

No detailed information on the timing of CAG and the strategy of PCI have been published recently for the German population. Early revascularisation by PCI is the guideline-based approach in patients with STEMI [14]. However, most OHCA patients show non-specific ECG changes after return of spontaneous circulation (ROSC). In these patients, the decision regarding the indication and timing of coronary angiography is more complex. The Angiography After Out-of-Hospital Cardiac Arrest Without ST-Segment Elevation (TOMAHAWK) [15] and the Coronary Angiography after Cardiac Arrest (COACT) trials [16] are the two available large-scale randomized multicentre trials comparing immediate versus delayed CAG in successfully resuscitated OHCA patients without ST-elevations on the ECG. Both, together with smaller randomized trials, showed no significant differences in clinical outcomes between the two strategies [15–17].

In our cohort, CAG was performed in 62.9% of patients. Immediate CAG was performed in three-quarters of patients. As only one-third of patients presented with ST-elevations after ROSC, this percentage seems rather high in the light of neutral trials in patients without ST-elevation. In total, 61.0% of patients undergoing CAG underwent PCI, corroborating the results of previous publications that coronary artery disease is the underlying problem in a relevant proportion of the patients. The high rate of culprit-only PCI (72.6%) might reflect the results of the CULPRIT-SHOCK trial, which showed a lower rate of death and severe renal failure in patients with infarct-related cardiogenic shock and multivessel disease who initially underwent culprit-only PCI compared with those who underwent immediate multivessel PCI [18]. Notably, despite the recommendation of current guidelines to use the radial access as the default strategy [19, 20], more than two-thirds of the procedures were performed via femoral access. According to the annual report of the GRR 2022, CAG was performed in 26.9%, which is much lower than in our cohort. Most likely, this is again explained by the selection of the centres. The GRR report does not provide detailed data on the timing of CAG, the rates, and the strategy of PCI [10].

### Targeted temperature management (TTM)

The optimal implementation of TTM and its influence on the neurological outcome are still controversially discussed. In 2021, the guideline of the European Resuscitation Council (ERC) recommended therapeutic hypothermia (TH) for all comatose patients after ROSC [16]. However, recent published data shows no significant difference in mortality or functional outcomes between TH with a target temperature of 33.0 °C and normothermia with fever prevention (≤ 37.7 °C) in adult OHCA patients with persistent coma after ROSC [21]. In 2022, the ERC guideline downgraded its recommendation for TH but recommended active fever prevention for at least 72 h [17]. The ESC 2023 ACS guidelines recommend continuous monitoring of core temperature and active fever prevention in OHCA patients with persistent coma, but do not give a statement on TH [15]. However, the before-mentioned recent data cannot be generalized for Germany because of various differences in patient characteristics (e.g. longer no-flow times, lower percentage of shockable rhythms, and lower bystander resuscitation rates in Germany) [22]. Furthermore, according to the most recent Cochrane systematic review and meta-analysis including all randomized controlled trials on TTM following OHCA, TH in the range of 32–34 °C as compared to normothermia or no temperature control was associated with improved neurologic outcomes after cardiac arrest [23]. This resulted in respective statements of national and international societies [24, 25]. The inconsistency of the data and the guideline recommendations is reflected in our data: TTM was performed in 44.5%. TH was applied in almost 87.0% of these patients, most likely because of the recommendations of the German national societies. Notably, there was a substantial variance in the mode of TTM application, i.e. invasive (device-based) vs. non-invasive. The rate of fever prevention (69.3%) was lower than could be expected due to the recommendations and in view of the fact that 87.4% of the patients were ventilated at admission. Most likely, this is due to an underreporting, since upon request, almost all recruiting centres stated to have a protocol for fever prevention.

### eCPR

The poor results of conventional CPR and the increased availability of percutaneously implantable mechanical circulatory support systems (ECLS, extracorporeal life support) has led to an increase in the rates of eCPR. In the small, single-centre ARREST trial conducted in a high-volume centre, a clear advantage of eCPR over conventional CPR was observed [26]. The Prague-OHCA trial was also a single-centre trial and compared an invasive approach including intra-arrest transport, eCPR, and immediate invasive assessment and treatment with a standard approach. The results regarding the primary endpoint (survival with good neurological outcome at 180 days) were neutral. The secondary endpoint, neurological recovery at 30 days, was observed significantly more often in the hyperinvasive group [27]. The most recently published randomized and only multicentre trial in this field is the INCEPTION trial conducted in 10 centres in the Netherlands. Herein, no improvement of survival with a favourable neurological outcome at 30 days was observed for eCPR [28]. The use of eCPR in G-CAR (11.0%) is higher compared to data from other registries. In a prospective registry conducted in Paris including more than 13,000 patients between 2011 and 2018, eCPR was performed in 4.0%. The GRR reported the use of ECLS in 8.3% of OHCA patients in 2021, without specifying the proportion of eCPR [29]. The higher use of eCPR in our registry most likely reflects the selection of centres. A major future task will be to understand whether there are subgroups of patients who might benefit from such a highly invasive, expensive, and resource-binding treatment. Besides the conduction of further randomized clinical trials, registries reflecting the real-world will help to provide answers.

### Outcomes

The observed in-hospital mortality was 70.5%; i.e., 29.5% of patients were discharged alive. According to the GRR 2022 report, 10.7% of the patients survived 30 days or were discharged alive [10]. However, the outcome data of the two registries are not comparable since the GRR reports total mortality rates, meaning that in contrast to G-CAR, the GRR also includes patients who are declared dead before arrival at the hospital. Notably, the survival rate of OHCA patients reaching the hospital in the large EuReCa TWO study was 26.4%, i.e. similar to our results [5]. Furthermore, there are more patients with respiratory/hypoxic and non-cardiac causes, such as trauma or drowning, in the GRR compared to G-CAR, which might lead to worse survival rates. Anoxic brain damage was the main cause of death and in more than half of the patients in hospital treatment was terminated, mainly because of brain damage or the patient's will. This underlines the need for an early and standardized neurological assessment and for a trained team taking care of the patients and relatives and discussing the patient’s (presumed) will in order to provide a medically and ethically correct treatment.

### Post-hospital phase

Psychosocial aspects such as health-related quality of life and psychopathological symptoms are important sequelae after OHCA [30]. As described previously (9), we are investigating several patient-reported outcomes such as quality of life, affective disorders, social reintegration, post-traumatic stress syndrome, as well as cognitive impairment by postal dispatch of standardized questionnaires and telephone interviews at 6 and 12 months. Publication of these results is planned separately.

### Limitations

G-CAR is a registry study with all well-known limitations of registries. However, registry studies are important to provide “real-world” data adding information to the results of randomized clinical trials and showing the extent to which they are implemented in clinical practice. Furthermore, registries are an important tool for quality control and are requested for certification as cardiac arrest centre. Another limitation is the above-mentioned centre bias with a focus on tertiary centres including many patients with underlying cardiac causes, especially ACS, impairing the generalizability of the data. Furthermore, the lack of information about the number of patients refusing participation might induce a bias towards the deceased patients. Last, missing values for specific parameters might induce a bias in the estimated results.

## Summary

G-CAR is a national multicentre registry of adult OHCA patients, providing detailed information with a focus on interventional treatment aspects. The results of the 16-month pilot phase are presented herein. The fact that the available evidence for OHCA patients is often scarce or contradictory underlines the usefulness of this first cardiologic resuscitation registry. Besides the national extension of G-CAR, assessment and publication of the patient-reported outcomes is planned.
